# Comparing symptom clusters in cancer survivors by cancer diagnosis: A latent class profile analysis

**DOI:** 10.1007/s00520-024-08489-0

**Published:** 2024-04-25

**Authors:** Lena J. Lee, Claire J. Han, Leorey Saligan, Gwenyth R. Wallen

**Affiliations:** 1 National Institutes of Health (NIH) Clinical Center, Bethesda, MD USA; 2 Ohio State University, College of Nursing, Columbus, OH USA; 3 National Institute of Nursing Research (NINR), Bethesda, MD USA; 4 National Institutes of Health (NIH) Clinical Center, Bethesda, MD USA

**Keywords:** Cancer survivors, Symptom clusters, Symptom management, Latent class analysis

## Abstract

**Purpose:**

Research on symptom clusters in oncology is progressing, but knowledge gaps remain. One question is whether the number and types of symptom subgroups (i.e., latent classes) differ based on cancer diagnosis. The purpose of this study was to: (1) identify and compare latent class subgroups based on four highly prevalent symptoms (pain, fatigue, sleep disturbance, and depression), and (2) examine the differences in sociodemographic and clinical factors in the identified latent classes across the seven cancer types (i.e., prostate, non-small cell lung, non-Hodgkin’s lymphoma, breast, uterine, cervical, and colorectal cancer).

**Methods:**

This study is a cross-sectional secondary analysis of data obtained from the My-Health study in partnership with four Surveillance, Epidemiology, and End Results (SEER) cancer registries located in California (two), Louisiana, and New Jersey. The sample included 4,762 cancer survivors 6-13 months following diagnosis of one of the seven cancer types mentioned. Latent class profile analysis was used.

**Results:**

Subjects were primarily young (59% age 21-64 years), Caucasian (41%), married/cohabitating (58%) and unemployed (55%). The number and types of symptom subgroups varied across these seven cancer populations: four-subgroups were the common in prostate, lung, non-Hodgkin’s lymphoma, and breast cancer survivors. Unmarried, low education, and unemployment status were associated with high risk of symptom burden across the cancer types.

**Conclusion:**

Identifying symptom subgroups by cancer diagnosis has the potential to develop innovative and effective targeted interventions in cancer survivors. Further research is needed to establish extensive knowledge in symptom clustering between treatment regimens, and short-term and long-term cancer survivors.

**Supplementary Information:**

The online version contains supplementary material available at 10.1007/s00520-024-08489-0.

## Introduction

It is well-established that individuals with cancer typically experience multiple symptoms [1], and pain, fatigue, sleep disturbance, and depression are common frequently concurrent symptoms reported by oncology patients [1, 2]. Recent literature in symptom management has emphasized a shift of focus from treating single symptoms to managing the dynamic nature of multiple symptom constellations, or symptom clusters [1]. A symptom cluster has been defined as two or more co-occurring symptoms that are related to each other. Symptom clusters are composed of stable groups of symptoms that are relatively independent of other clusters, and they may share underlying mechanisms and/or outcomes [3]. There is a growing body of evidence that symptom clusters might have significant effects on the health outcomes of patients with cancer, such as quality of life and functional status, often in a multiplicative rather than additive manner [4–6].

Although the identification of symptom clusters can result in effective symptom management interventions, gaps in symptom cluster research can be found in conceptual, methodological, and analytical issues [1, 7]. The conceptual issue relates to the manner in which symptom clusters are defined. Symptom clusters have been identified using two approaches: the empiric or “de novo” identification of symptom clusters, and the identification of subgroups of people based on their experiences with a specific symptom cluster (i.e., latent classes). Methodological issues include whether symptoms should be measured with unidimensional instruments assessing several symptoms or by several instruments, each focusing on more than one dimension of the same symptom. The analytical level is arguably the best manner of statistically identifying the existence of a symptom cluster [1]. The large number of measurements and statistical methods that have been used to identify symptom clusters, with little guidance regarding the justification of the choice of method, is a major barrier to the conceptual validity and clinical utility of statistically derived symptom clusters [1, 4].

Given the lack of consensus on conceptual, methodological, and analytical issues of symptom cluster research, numerous unanswered questions remain [1, 4]. One question is whether the number and types of symptom clusters differ depending on the cancer diagnosis. Using a heterogeneous sample can be challenging to determine if the symptom cluster presentation is inconsistent for a specific type of cancer. In addition, the extent to which the reported clusters are dependent on the cancer diagnoses is unclear. The use of different measurement tools and analytical methods to evaluate symptoms may also result in a different construction of symptom clusters. To compensate for heterogeneous samples and the use of inconsistent methodology, research is needed to evaluate for differences in the number and types of symptom clusters across cancer diagnoses using the same assessment tools and statistical methods. Consistent identification of symptom clusters is key to the development of targeted and tailored interventions for symptom clusters.

To date, relatively few studies have evaluated for symptom clusters across cancer diagnosis [8–10]. Dong and colleagues [8] investigated the consistency of symptom composition in patients with advanced cancer using three different statistical approaches (i.e., principal component analysis, exploratory factor analysis, cluster analysis) and demonstrated that symptom clusters were relatively consistent across five primary cancer sites (i.e., prostate, breast, lung, colorectal, myeloma). However, the patient samples used have been insufficient in size to allow comparison of cluster composition among cancer types. In addition, there have only been a few published studies that compared symptom clusters using the same statistical method [9, 10]. Harris and colleagues [9] identified symptom clusters using exploratory factor analysis in 1,329 oncology patients with four different cancer types (i.e., breast, gastrointestinal, gynecological, and lung cancers). In this study, psychological, weight gain and gastrointestinal clusters were stable across cancer types, while respiratory and hormonal clusters were unique to specific cancer diagnoses. Another study [10] assessing differences in symptom clustering across 1,330 survivors of five types of cancer (i.e., colorectal, breast, ovarian, thyroid, and hematological cancers) using the network analysis found that fatigue was consistently the most central symptom in the network with other symptoms (e.g., emotional symptoms, pain) across all cancer types.

However, previous studies in symptom clusters aforementioned above [8–10] have small sample size, inconsistent symptom measures across the studies, and variables-centered approaches (i.e., factor analysis). These limitations may hinder to identify distinct symptom phenotypes vary by different cancer types. The latent class model approach as a person-centered approach is a statistical method for identifying unobserved (i.e., latent) subgroups. The latent class analysis is beneficial for symptom research, where data often include heterogeneous groups of individuals with cancer experiencing multiple symptoms, and for identifying distinct subgroups, particularly with a group of common symptoms or a pre-defined symptom cluster, based on their symptoms [11, 12]. Although latent class modeling was used in a few studies in cancer survivors to identify symptom clusters [13–15], no published symptom cluster study using latent class analysis has compared distinct symptom subgroups across different cancer types. Comprehensive and representative samples of cancer survivors using the latent class approach in conjunction with well-validated and reliable patient-reported outcomes might be helpful in clarifying how subgroups of survivors may differ across different cancer diagnosis based on a symptom cluster. The purposes of this study were (1) to identify and compare latent class subgroups based on four highly prevalent symptoms (i.e., pain, fatigue, sleep disturbance, and depression) and (2) to investigate the differences in sociodemographic and clinical factors in the identified latent classes across seven populations of cancer survivors (i.e., prostate cancer, non-small cell lung cancer, non-Hodgkin’s lymphoma [NHL], breast cancer, uterine cancer, cervical cancer, and colorectal cancer).

## Methods

### Study cohort

This study was a cross-sectional secondary analysis of data from the Measuring Your Health (MY-Health) study, a prospective cohort study. In the parent study, data were collected to evaluate the health and well-being of a diverse cohort of individuals with cancer [16]. Participants in the MY-Health study were recruited through four Surveillance, Epidemiology, and End Results (SEER) registries located in California (two), Louisiana, and New Jersey between 2010 and 2012. Participants were eligible for the parent study if they (a) were 21-84 years old at the time of initial diagnosis of one of seven types of cancer (i.e., prostate cancer, non-small cell lung cancer, NHL, female breast cancer, uterine cancer, cervical cancer, and colorectal cancer); and (b) could read and speak English, Spanish, or Mandarin. Details of the study design, study procedures, and participant descriptions have been reported elsewhere [16]. This study was approved by the institutional review boards for each participating SEER site and Georgetown University (Washington, DC).

The survey was completed by 5,506 individuals with cancer. For this analysis, we restricted eligibility to individuals diagnosed with cancer within the previous six to thirteen months. Of 5,506 individuals with cancer, a total of 4,762 participants were included following the exclusion of 409 participants diagnosed thirteen or more months prior, 333 participants who had died during the survey period, and two participants with incomplete responses.

### Measures

Symptoms were measured using the Patient Reported Outcomes Measurement Information System (PROMIS^®^) measures extensively validated in individuals with cancer [17]. The MY-Health study administered PROMIS^®^ custom short forms assessing pain interference (10 items), fatigue (14 items), sleep disturbance (10 items), and depression (10 items). The PROMIS^®^ measures are scored on a 5-point Likert-type scale from 1 (*never*) to 5 (*always*). Higher scores indicate higher symptom severity. The PROMIS^®^ measures are calibrated and standardized to a T-score metric, with a mean of 50 and a standard deviation of 10 centered on the general United States population. The measure offers clinically relevant symptom thresholds and are as follows [18, 19].

- Pain: <50 normal; 50-59 mild; 60-69 moderate; ≥70 severe
- Fatigue: <50 normal; 50-54 mild; 55-74 moderate; ≥75 severe
- Sleep disturbance: <45 normal, 45-54 mild; 55-59 moderate: ≥60 severe
- Depression: <55 normal, 55-64 mild; 65-74 moderate; ≥75 severe

In this study, the internal consistency of the instrument was high (Cronbach’s alpha for pain interference = 0.98; fatigue = 0.96; sleep disturbance = 0.95; and depression = 0.97).

### Statistical analysis

Latent class model approach, a type of finite mixture model, was used to divide our sample into unobserved (i.e., latent) homogeneous subgroups, which are called classes, using multiple observed variables. In the latent class analysis, categorical variables are typically used. When the indicators are continuous variables, it is termed latent class profile analysis (LCPA) [12]. In this study, seven separate LCPA (based on the seven cancer diagnoses) were conducted on the four symptom variables (i.e., pain, fatigue, sleep disturbance, depression). Estimation was carried out with the robust maximum-likelihood and Expectation-Maximization algorithms [12]. Statistical fit indices were used to both evaluate model fit and to determine the final number of latent classes. The model that fits the data best was selected by a combination of the following criteria: (1) the lowest Akaike information criterion (AIC); (2) the lowest Bayesian information criterion (BIC); (3) the lowest Vuong-Lo-Mendell Rubin likelihood ratio test (VLMR); (4) the lowest parametric bootstrapped likelihood ratio test (BLRT); and (5) entropy to be 0.80 or greater [12]. The latent classes were named based on established symptom cut points [18, 19]. Multivariate analysis of variance (MANOVA) was performed to analyze the differences in symptom severity among the latent classes. In addition, sociodemographic and clinical characteristics differ by latent class subgroups per cancer type were examined using Chi-square test. Mplus Version 8.6 was used for LCPA [20]. Other analyses were conducted with SPSS Version 28.0 for Windows [21].

## Results

### Sociodemographic and clinical characteristics of subjects

Data for 4,762 participants were examined in this analysis (Table 1). The participants were predominantly younger at diagnosis (21-64 years) (63.5%), female (60.3%), White (53.1%), non-Hispanic (80.3%), and married/cohabiting (58.9%). In terms of clinical characteristics, the majority of participants had cancer stages I and II (73.2%) and were primarily treated with surgery (69.3%).

**Table 1:** Sociodemographic and clinical characteristics of the study sample and by cancer diagnosis

Variables	N (%)							
	Total (*N* =4,762)	Prostate (*N*=1,060)	Lung (*N*=526)	NHL (*N*=390)	Breast (*N*=1,500)	Uterine (*N*=354)	Cervical (*N* = 130)	Colorectal (*N*=802)
Age at diagnosis (years)								
21- 49	1167 (24.5)	68 ( 6.4)	44 ( 8.4)	114 (29.2)	611 (40.7)	83 (23.4)	93 (71.5)	154 (19.2)
50- 64	1859 (39.0)	485 (45.8)	209 (39.7)	129 (33.1)	514 (34.3)	187 (52.8)	24 (18.5)	311 (38.8)
65 or older	1736 (36.5)	507 (47.8)	273 (51.9)	147 (37.7)	375 (25.0)	84 (23.7)	13 (10.0)	337 (42.0)
Sex								
Male	1889 (39.7)	1060 (100.0)	245 (46.6)	200 (51.3)	0 ( 0.0)	0 ( 0.0)	0 ( 0.0)	383 (47.8)
Female	2873 (60.3)	0 ( 0.0)	281 (53.4)	190 (48.7)	1500 (100.0)	354 (100.0)	130 (100.0)	419 (52.2)
Race								
White	2529 (53.1)	530 (50.1)	365 (69.4)	259 (66.4)	715 (47.8)	178 (50.3)	73 (56.2)	409 (51.0)
Black	998 (21.0)	301 (28.5)	83 (15.8)	50 (12.8)	288 (19.2)	69 (19.5)	23 (17.7)	184 (22.9)
Asian	836 (17.6)	142 (13.4)	50 ( 9.5)	50 (12.8)	367 (24.5)	64 (18.1)	17 (13.1)	146 (18.2)
Other	394 ( 8.3)	84 ( 7.9)	28 ( 5.3)	31 ( 7.9)	128 ( 8.5)	43 (12.1)	17 (13.1)	63 (7.9)
Ethnicity								
Hispanic	939 (19.7)	212 (20.0)	51 ( 9.7)	83 (21.3)	295 (19.7)	96 (27.1)	45 (34.6)	157 (19.6)
Non-Hispanic	3823 (80.3)	848 (80.0)	475 (90.3)	307 (78.7)	1205 (80.3)	258 (72.9)	85 (65.4)	645 (80.4)
Marital status								
Married/cohabiting	2780 (58.9)	761 (72.5)	303 (57.8)	224 (58.0)	835 (56.2)	166 (47.3)	57 (44.5)	434 (54.8)
Not married^a^	1936 (41.1)	289 (27.5)	221 (42.2)	162 (42.0)	650 (43.8)	185 (52.7)	71 (55.5)	358 (45.2)
Education level								
≤ High school	1708 (36.3)	376 (35.9)	228 (43.8)	137 (35.7)	443 (29.9)	122 (35.1)	59 (46.5)	343 (43.4)
Some college	1549 (33.0)	332 (31.7)	192 (36.9)	121 (31.5)	493 (33.2)	116 (33.3)	43 (33.9)	252 (31.9)
Undergraduate degree or greater	1442 (30.7)	339 (32.4)	100 (19.2)	126 (32.8)	547 (36.9)	110 (31.6)	25 (19.7)	195 (24.7)
Employment status^b^								
Working	2138 (45.5)	459 (43.4)	118 (22.8)	184 (48.2)	843 (56.9)	170 (48.2)	66 (51.2)	298 (38.2)
Not Working	2562 (54.5)	598 (56.6)	399 (77.2)	198 (51.8)	638 (43.1)	183 (51.8)	63 (48.8)	483 (61.8)
Stage at diagnosis								
I	1810 (39.6)	272 (26.5)	185 (36.3)	126 (34.0)	693 (48.3)	276 (79.3)	70 (57.4)	188 (24.6)
II	1537 (33.6)	591 (57.6)	73 (14.3)	80 (21.6)	557 (38.8)	14 ( 4.0)	12 ( 9.8)	210 (27.5)
III	786 (17.2)	109 (10.6)	138 (27.1)	52 (14.0)	154 (10.7)	45 (12.9)	29 (23.8)	259 (33.9)
IV	442 ( 9.7)	54 ( 5.3)	113 (22.2)	113 (30.5)	32 ( 2.2)	13 ( 3.7)	11 ( 9.0)	106 (13.9)
Cancer treatment history^c^								
Surgery	3302 (69.3)	483 (46.5)	291 (55.7)	69 (18.0)	1379 (91.9)	311 (88.6)	82 (64.1)	687 (87.6)
Chemotherapy	2182 (45.8)	69 ( 6.8)	307 (59.0)	290 (75.3)	891 (59.4)	88 (25.3)	73 (56.6)	464 (58.7)
Radiation therapy	1936 (40.7)	408 (39.0)	225 (43.1)	101 (26.1)	875 (58.3)	115 (33.0)	78 (60.9)	134 (17.1)

Numbers may not sum to total due to missing data

Abbreviations: *NHL* non-Hodgkin lymphoma, *SD* standard deviation

^a^ Not married = never married, separated or divorced, widowed

^b^ Employment status = working (employed, homemaker, student); not working (retired, disability, unemployed)

^c^ Multiple response questions

### Identification of latent class subgroups based on symptoms by cancer diagnosis

**Fig. 1:**
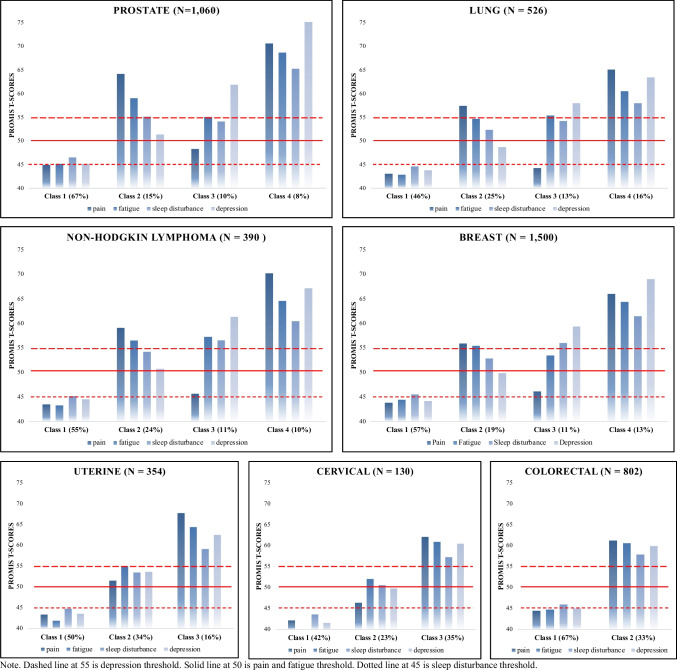
Differences in Symptoms among the Latent Classes in Cancer Survivors (*N* = 4,762)

The results of statistical fit indices for the candidate models are shown in Table 2. As summarized in Table 3 and illustrated in Fig. 1, four distinct classes were identified in prostate, non-small cell lung, and breast cancer, and NHL survivors; three classes were discovered in uterus and cervical cancer survivors; and two classes were identified in colorectal cancer survivors based on symptom severity and types of symptoms.

**Table 2:** Model fit information for LCPA models fit to data in cancer survivors

Class	AIC	BIC	Entropy	VLMR^a^	BLRT^a^
Prostate					
2	28767.642	28832.201	0.943	*p* < .001	*p* < .001
3	28330.122	28419.511	0.929	*p* =0.0013	*p* < .001
4^b^	28005.529	28119.747	0.923	*p* =0.0098	*p* < .001
5	27777.003	27916.052	0.927	*p* =0.0664	*p* < .001
Lung					
2	14325.912	14381.361	0.867	*p* < .001	*p* < .001
3	14163.871	14240.647	0.805	*p* =0.0189	*p* < .001
4^b^	14015.976	14114.078	0.853	*p* =0.0149	*p* < .001
5	13973.228	14092.657	0.857	*p* =0.2992	*p* < .001
Non-Hodgkin lymphoma					
2	42453.566	42522.638	0.882	*p* < .001	*p* < .001
3	41989.507	42085.145	0.823	*p* < .001	*p* < .001
4^b^	41724.690	41846.894	0.861	*p* =0.0056	*p* < .001
5	41580.397	41729.167	0.861	*p* =0.1403	*p* < .001
Breast					
2	42453.566	42522.638	0.882	*p* < .001	*p* < .001
3	41989.507	42085.145	0.823	*p* < .001	*p* < .001
4^b^	41724.690	41846.894	0.861	*p* =0.0056	*p* < .001
5	41580.397	41729.167	0.861	*p* =0.1403	*p* < .001
Uterine					
2	28330.122	28419.511	0.929	*p* =0.0013	*p* < .001
3^c^	28005.529	28119.747	0.923	*p* =0.0098	*p* < .001
4	27777.003	27916.052	0.927	*p* =0.0664	*p* < .001
Cervical					
2	28330.122	28419.511	0.929	*p* =0.0013	*p* < .001
3^c^	28005.529	28119.747	0.923	*p* =0.0098	*p* < .001
4	27777.003	27916.052	0.927	*p* =0.0664	*p* < .001
Colorectal					
2^d^	21862.392	21923.324	0.888	*p* < .001	*p* < .001
3	21641.517	21725.885	0.810	*p* =0.4891	*p* < .001

^a^ Chi-square statistic for the VLMR and the BLRT, when non-significant (*p* > .05), the VLMR and the BLRT test provide evidence that K-1 class model fits the data better than the K-class model

^b^ Four-class model was selected based on its having a smaller BIC than the three-class model and nonsignificant VLMR in the five-class model

^c^ Three-class model was selected based on its having a smaller BIC than the two-class model and nonsignificant VLMR in the four-class model

^d^ Two-class model was selected based on nonsignificant VLMR in the three-class model

*AIC* Akaike information criterion, *BIC* Bayesian information criterion, *BLRT* Bootstrapped likelihood ratio test, *LCPA* latent class profile analysis, *VLMR* Vuong-Lo-Mendell-Rubin

**Table 3:** Differences in Severity of Symptoms among the Latent Classes in Cancer Survivors

Variable	Mean (SD)				*p* value
	Class 1	Class 2	Class 3	Class 4	
Prostate (*N*=1,060)					
	*WNL* (*n*=711, 67%)	*Fatigue/SD/Pain* (*n*=158, 15%)	*Fatigue/SD/Depression* (*n*=108, 10%)	*All Symptoms* (*n*=83, 8%)	
Pain	44.86 (3.62)	64.09 (5.98)	48.22 (5.26)	70.48 (6.94)	< .001
Fatigue	45.07 (5.88)	58.95 (7.89)	54.96 (7.05)	68.52 (7.77)	< .001
Sleep disturbance	46.43 (7.93)	55.02 (9.47)	54.02 (8.21)	65.20 (8.55)	< .001
Depression	45.08 (3.16)	51.28 (6.56)	61.78 (5.87)	75.11 (7.76)	< .001
Non-small Cell Lung (*N*=526)					
	*WNL* (*n*=244, 46%)	*Fatigue/SD/Pain* (*n*=129, 25%)	*Fatigue/SD/Depression* (*n*=68, 13%)	*All Symptoms* (*n*=85, 16%)	
Pain	42.98 (4.98)	57.37 (5.42)	45.30 (6.49)	63.35 (7.25)	< .001
Fatigue	42.78 (6.82)	54.62 (5.64)	55.31 (8.40)	60.45 (8.39)	< .001
Sleep disturbance	44.54 (8.01)	52.28 (8.25)	54.14 (9.00)	58.92 (8.95)	< .001
Depression	43.71 (5.07)	48.61 (6.03)	57.90 (8.10)	63.33 (9.23)	< .001
Non-Hodgkin lymphoma (*N*= 390)					
	*WNL* (*n*=216, 55%)	*Fatigue/SD/Pain* (*n*=92, 24%)	*Fatigue/SD/Depression* (*n*=42, 11%)	*All Symptoms* (*n*=40, 10%)	
Pain	43.40 (2.87)	58.99 (4.24)	45.59 (4.09)	70.17 (3.98)	< .001
Fatigue	43.18 (5.91)	56.41 (6.37)	57.16 (5.94)	64.54 (6.62)	< .001
Sleep disturbance	45.14 (8.03)	54.14 (8.60)	56.51 (8.22)	60.41 (8.67)	< .001
Depression	44.46 (4.71)	50.69 (7.20)	61.24 (7.49)	67.13 (9.68)	< .001
Breast (*N*=1,500)					
	*WNL* (*n*=864, 57%)	*Fatigue/Pain/SD* (*n*=282, 19%)	*SD/Fatigue/Depression* (*n*=161, 11%)	*All Symptoms* (*n*=193, 13%)	
Pain	43.77 (4.31)	60.35 (5.26)	46.10 (4.90)	65.99 (5.89)	< .001
Fatigue	44.39 (6.48)	55.36 (8.09)	53.40 (8.32)	64.32 (6.98)	< .001
Sleep disturbance	45.45 (7.93)	52.79 (8.49)	55.96 (8.37)	61.42 (8.33)	< .001
Depression	44.10 (3.68)	49.80 (5.96)	59.26 (6.16)	68.96 (6.75)	< .001
Uterine (*N*=354)					
	*WNL* (*n*=178, 50%)	*Fatigue/SD/Pain* (*n*=119, 34%)	*All Symptoms* (*n*=57, 16%)		
Pain	43.41 (3.68)	51.57 (6.87)	67.80 (5.18)		< .001
Fatigue	41.98 (4.24)	55.00 (5.72)	64.44 (5.78)		< .001
Sleep disturbance	44.75 (7.56)	53.50 (9.10)	59.17 (8.70)		< .001
Depression	43.60 (4.25)	53.70 (8.68)	62.57 (9.96)		< .001
Cervical (*N*=130)					
	*WNL* (*n*=55, 42%)	*Fatigue/SD* (*n*=30, 23%)	*All Symptoms* (*n*=45, 35%)		
Pain	42.24 (2.32)	46.41 (5.44)	62.19 (5.70)		< .001
Fatigue	40.01 (3.34)	52.10 (4.07)	60.95 (4.10)		< .001
Sleep disturbance	43.61 (7.38)	50.59 (8.21)	57.30 (8.76)		< .001
Depression	41.64 (2.56)	49.85 (6.41)	60.55 (7.65)		< .001
Colorectal (*N*=802)					
	*WNL* (*n*=534, 67%)	*All Symptoms* (*n*=268, 33%)			
Pain	44.48 (5.42)	61.30 (7.38)			< .001
Fatigue	44.77 (6.87)	60.66 (6.19)			< .001
Sleep disturbance	46.04 (8.21)	57.91 (8.45)			< .001
Depression	45.04 (5.34)	59.96 (9.75)			< .001

*SD* sleep disturbance, *SD* standard deviation, *WNL* within normal limits

Reference PROMIS t scores have been established for the cancer patient subpopulation (i.e., pain: <50 normal; 50–59 mild; 60–69 moderate; ≥70 severe; fatigue: <50 normal; 50–54 mild; 55–74 moderate; ≥75 severe; sleep disturbance: <45 normal; 45–54 mild; 55–59 moderate; ≥ 60 severe; depression: <55 normal; 55–64 mild; 65–74 moderate; ≥75 severe)

#### Four-class solution

##### Prostate

Class 1 (67%), labeled *Within Normal Limits (WNL),* was characterized by all four symptoms within normal limits, based on established symptom cut points [18, 19]. Class 2 (15%) was characterized by mild pain with moderate fatigue and sleep disturbance, but no elevated depression (within normal limits), and was labeled *Fatigue/sleep disturbance (SD)/Pain.* Class 3 (10%) was characterized by mild sleep disturbance and depression with moderate fatigue, but pain was within normal limits, and was labeled as *Fatigue/SD/Depression.* Class 4 (8%), labeled *All Symptoms,* was characterized by moderate fatigue and sleep disturbance with severe pain and depression.

##### Non-small cell lung

Class 1 (46%), labeled *WNL,* was characterized by all four symptoms within normal limits. Class 2 (25%) was characterized by mild pain, fatigue, and sleep disturbance, but depression was within normal limits, and was labeled *Fatigue/SD/Pain.* Class 3 (13%) was characterized by mild sleep disturbance and depression, with moderate fatigue, but pain was within normal limits, and was labeled by *Fatigue/SD/Depression.* Class 4 (16%), labeled *All Symptoms,* was characterized by mild depression, with moderate pain, sleep disturbance, and fatigue.

##### NHL

Class 1 (55%), labeled *WNL,* was characterized by all four symptoms within normal limits. Class 2 (24%) was characterized by mild pain and sleep disturbance, with moderate fatigue, but depression was within normal limits, and was labeled *Fatigue/SD/Pain.* Class 3 (11%) was characterized by mild depression, with moderate fatigue and sleep disturbance, but pain was within normal limits, and was labeled by *Fatigue/SD/Depression.* Class 4 (10%), labeled *All Symptoms,* was characterized by moderate fatigue, sleep disturbance and depression, with severe pain.

##### Breast

Class 1 (57%), labeled *WNL,* was characterized by all four symptoms within normal limits. Class 2 (19%) was characterized by mild sleep disturbance, with moderate pain and fatigue, but depression was within normal limits, and was labeled *Fatigue/Pain/SD.* Class 3 (11%) was characterized by mild fatigue and depression, with moderate sleep disturbance, but pain was within normal limits, and was labeled by *SD/Fatigue/Depression.* Class 4 (13%), labeled *All Symptoms* was characterized by moderate pain, fatigue, and depression, with severe sleep disturbance.

#### Three-class Solution

##### Uterine

Class 1 (50%), labeled *WNL,* was characterized by all four symptoms within normal limits. Class 2 (34%) was characterized by mild pain and sleep disturbance, with moderate fatigue, but depression was within normal limits, and was labeled *Fatigue/SD/Pain.* Class 3 (16%) was characterized by mild depression, with moderate pain, fatigue, and sleep disturbance, and was labeled by *All Symptoms.*

##### Cervical

Three distinct classes of cervical cancer survivors were identified. Class 1 (42%), labeled *WNL,* was characterized by all four symptoms within normal limits. Class 2 (23%) was characterized by mild fatigue and sleep disturbance, but pain and depression were within normal limits, and was labeled *Fatigue/SD.* Class 3 (35%) was characterized by mild depression, with moderate pain, fatigue and sleep disturbance, and was labeled by *All Symptoms.*

#### Two-class Solution

##### Colorectal

Two distinct classes of cervical cancer survivors were identified. Class 1 (67%), labeled *WNL,* was characterized by all four symptoms within normal limits. Class 2 (33%) was characterized by mild depression, with moderate pain, fatigue, and sleep disturbance, and was labeled by *All Symptoms.*

### Sociodemographic and clinical characteristics of the latent class subgroups

The sociodemographic and clinical characteristics of the latent classes in seven cancer survivors are shown in Table 4 and Supplementary Table S1. Among the sociodemographic factors examined, there were statistically significant differences in marital status, education level, and employment status across the identified latent classes in all seven cancer survivors. Class 1 (*WNL*) had a higher proportion of being married, survivors with an undergraduate degree or greater, and individuals who are working among identified latent classes than the symptomatic classes (Class 2, Class 3, and Class 3). In prostate, lung, breast, and colorectal cancers, age at diagnosis was associated with latent classes and younger age was associated with symptomatic classes than Class 1. Both race and ethnicity were associated with latent classes in NHL, breast, and colorectal cancers.

**Table 4:** Differences in characteristics of each of the latent classes in cancer survivors

Variables	Prostate	Lung	NHL	Breast	Uterine	Cervical	Colorectal
Age at diagnosis	•	•		•			•
Sex	N/A			N/A	N/A	N/A	
Race	•		•	•	•		•
Ethnicity			•	•	•	•	•
Marital status	•	•	•	•	•	•	•
Education level	•	•	•	•	•	•	•
Employment status	•	•	•	•	•	•	•
Stage at diagnosis	•			•			•
Surgery				•			•
Chemotherapy			•	•			•
Radiotherapy	•						•

Abbreviations: *N/A* Not applicable, *NHL* non-Hodgkin lymphoma

The bullet points indicate factors significantly different in the identified latent classes among the cancer survivors (*p* < .05)

Among the clinical factors examined, cancer treatment history was associated with latent classes in breast and colorectal cancers (surgery), chemotherapy (NHL, breast and colorectal cancers), and radiotherapy (prostate and colorectal cancers). Of note, all three cancer treatments (surgery, chemotherapy, and radiotherapy) were associated with latent classes in colorectal cancer.

## Discussion

To our knowledge, this study was the first to assess and compare distinct symptom subgroups based on symptoms by seven cancer diagnoses (i.e., prostate, non-small cell lung, NHL, breast, uterine, cervical, and colorectal cancer) in a large sample of cancer survivors using LCPA and a validated symptom measure (PROMIS^®^), ensuring reliable representations of distressing symptoms common or distinct to different cancers. Analyzing across cancers may unveil shared underlying mechanisms for more targeted interventions to pro-inflammatory status in cancers. Addressing shared inflammatory mechanisms could lead to targeted interventions.

Our study showed variability in the number and types of latent class subgroups based on the specific cancer diagnosis, indicating different symptom experiences among these groups. In prostate, lung, NHL, and breast cancers, four distinct latent classes of patients were identified (*WNL, Fatigue/SD/Pain, Fatigue/SD/Depression,* and *All Symptoms*). Fewer distinct symptom patterns were identified in uterine and cervical cancer (three latent classes) and colorectal cancer (two latent classes) compared to the previously mentioned cancer types. These findings have important implications for the management and care of patients across different cancer types. Common latent class subgroups across these seven cancer populations are *WNL* and *All Symptoms* groups. The most common four latent classes identified in prostate, lung, NHL, and breast cancers are consistent with previous literature. Our study findings in lung cancer different differ from a previous study on lung cancer survivors [22]. In a study of 378 lung cancer survivors based on pain, fatigue, sleep disturbance, depression, and cognitive impairment, all low and all high-symptom groups were identified [22]. These differences could be due to different sample sizes and instruments compared to our study. Despite fatigue, depression, anxiety, and sleep disturbance are prevalent symptoms in prostate cancer [23] and NHL [24], there is no previous study identified that specifically analyzes latent classes based on symptoms in prostate cancer and NHL. Therefore, our study shed light on the prevalence of these common symptom subgroups (i.e., *Fatigue/SD/Pain, Fatigue/SD/Depression*, and *All Symptoms*) in prostate cancer and NHL populations.

We found pain with fatigue and sleep disturbances and depression with fatigue and sleep disturbances were common latent classes across the cancer types, specifically for prostate, lung, NHL, and breast cancers. In 84 cancer patients with multiple cancer diagnoses, pain predicted fatigue and sleep disturbances and sleep disturbances mediated the link of pain with fatigue [24]. Among four common types of latent classes across prostate, lung, NHL, and breast cancers in our study, pain, and depression were associated with fatigue and sleep disturbances. Numerous studies in the literature have indicated a robust correlation between depression, fatigue, and sleep disturbances in cancer survivors [25–27]. Cancer-related fatigue is a distressing and persistent symptom frequently experienced by cancer survivors, often resulting from disrupted sleep patterns [26, 27]. The prevalence of fatigue and depression in cancer patients has been extensively researched, with a significant number of survivors reporting elevated depressive symptoms and experiencing fatigue [25]. Additionally, sleep disturbances are common among cancer patients, and studies have shown that these symptoms tend to co-occur, implying a potential shared underlying mechanisms such as proinflammatory cytokines [26]. While causal pathways for whether pain or depression predicts fatigue and sleep disturbances or vice versa are unknown, managing these interconnected symptoms is crucial for enhancing the quality of life and overall well-being of cancer survivors. Our findings showing pain or depression as a distinguishable factor of two latent classes (*Fatigue/SD/Pain* and *Fatigue/SD/Depression*) suggest addressing either pain or depression may help to alleviate co-occurring fatigue and sleep disturbances and improve the overall health of cancer survivors.

In uterine cancer survivors, *Fatigue/SD/Pain* symptom cluster was identified in our study. Pain is one of the most distressing and prevalent symptoms for women with uterine cancer [27]. Furthermore, a 24-month longitudinal study of gynecologic cancer patients found that pain persisted for up to 6-month post-cancer treatments [28]. Of note, women with gynecologic cancers with pain have reported subsequent psychological distress such as depression, anxiety, and fatigue [29]. Inflammation may play a significant role in pain experiences among uterine cancer survivors. Pain sensitization and perpetuation of symptoms are linked to cytokines, which activate both peripheral and central nervous system pathways. These mechanisms involve increased stimulation of the autonomic nervous system, cytokine release by brain glia, and localized and systemic actions of prostaglandins. Proinflammatory cytokines found in the bloodstream have been associated with pain symptoms in various populations, including those with chronic pain disorders and cancer [28]. Therefore, uterine cancer survivors may face an increased risk of inflammation-related pain due to the secretion of pro-inflammatory biomarkers, such as interleukin (IL)-6, by uterine tumors. Furthermore, proinflammatory cytokines are released in response to tissue damage from treatments like chemotherapy, radiation therapy, and surgery. Uterine cancer patients have reported various types of pain such as uterine cramps, pelvic pressure, and abdominal pain [30]. Therefore, types of pain experiences will be further investigated in future studies to better manage pain and pain-related symptom subgroups.

In our study, we found two latent classes (*WNL* and *All Symptoms*) in colorectal cancer survivors. Gastrointestinal symptom toxicities such as diarrhea, constipation, abdominal pain, bloating, nausea, and fecal leakage, after cancer treatments are prevalent and severe in colorectal cancer survivors, compared to non-gastrointestinal cancer types [31, 32]. Colorectal cancer survivors with high gastrointestinal symptoms also reported high psychoneurological symptoms [31–33]. Thus, further research to identify latent classes including gastrointestinal symptoms is warranted to better capture the complex symptom experiences in colorectal cancer survivors.

In our second aim, we investigated the relationships of sociodemographic and clinical factors with latent classes by cancer diagnosis to understand better the potential contributing factors of distinct latent classes that differ by cancer diagnosis. The research findings confirm that symptom experiences may differ across the various cancer types due to cancer-related specific factors such as cancer sites, cancer stages, as well as different cancer treatment regimens (e.g., surgery, chemotherapy, radiotherapy). Furthermore, our results indicating younger age groups with higher symptom burdens are consistent with previous findings [34]. Younger cancer survivors may experience a higher symptom burden compared to older patients due to their additional life responsibilities and potentially higher resilience levels. Younger individuals often juggle work, family, and caregiving responsibilities, which can exacerbate the impact of cancer-related symptoms [34]. Older patients, on the other hand, may have fewer external stressors and greater acceptance of their health conditions, leading to a perceived lower symptom burden. However, it is essential to consider individual variations in symptom experiences and coping mechanisms across different age groups. Our study findings align with previous research indicating a potentially high symptom burden among cancer survivors with unfavorable social determinants of health (SDOH) status, such as racial/ethnic minorities, low education, low-income status, or poor social support [35]. These factors may contribute to disparities in health outcomes and healthcare access, leading to poorer health literacy and exacerbating symptom experiences [36]. Addressing these disparities is crucial for improving the overall well-being and quality of life of cancer survivors [35].

### Implications for clinical practice and further research

Based on our research findings, clinicians should consider that addressing one symptom in isolation given a specific cancer diagnosis may not be sufficient to manage a patient's overall symptom burden. Instead, a holistic approach is needed to comprehensively manage distinct subgroups based on symptoms. Furthermore, approximately 33-58% of cancer survivors in our study experience moderate-to-severe symptom burden, which significantly affects their quality of life. Clinicians should be attentive to these individuals and proactively identify patients with high symptom burdens. Symptom interventions need to be multi-faceted, addressing multiple symptoms simultaneously. For example, lifestyle interventions that incorporate improved sleep hygiene, exercise, and nutrition may positively impact sleep, fatigue, pain, and depression levels, by adopting an integrated approach. The similarities and differences in patterns of symptom subgroups observed in this study raise important theoretical and practical considerations that warrant further investigation. Lastly, understanding sociodemographic and cancer-specific factors can aid in tailoring interventions to meet the diverse needs related to symptom management, in particular, underserved cancer survivors.

### Limitations

Our study has several limitations. Our study might have focused on specific seven cancer types or populations (e.g., Whites, female predominant samples), which could limit the generalizability of the findings to other cancer types or patient groups. The heterogeneity of cancer types and treatment regimens might impact symptom experiences differently in various patient populations. Secondly, the use of a cross-sectional design might limit the ability to establish causality and track the changes in symptom subgroups over time. Longitudinal studies would provide a more comprehensive understanding of the dynamic nature of symptom experiences in cancer survivors. Third, we used subjective symptom measures, thus symptom reports might vary based on individual perceptions and reporting biases. Some symptoms may be underreported or overlooked, affecting the accuracy of the identified symptom subgroups. The selected symptom domains for analysis might not capture the full spectrum of symptoms experienced by cancer survivors with various types of cancer diagnoses. Additional symptoms relevant to specific cancer types or treatments might be omitted, potentially influencing the psychoneurological symptoms (e.g., respiratory symptoms in lung cancer, gastrointestinal symptoms in colorectal cancer, and pelvic pain in uterine cancer).

## Conclusions

This study explored and compared latent classes based on pain, fatigue, depression, and sleep disturbances among cancer survivors across multiple cancer diagnoses. Our findings add to prior results that numbers and types of symptom subgroups are relatively similar across the four cancer types (prostate, lung, NHL, and breast), and the variation exists in the number of symptom subgroups in uterine, cervical, and colorectal cancers. Cancer-specific factors were significant factors in distinguishing latent classes, while younger age and poor SDOH status were common factors contributing to high symptom burden across seven cancer types. The findings of this study guide future research such as the development of individualized symptom management to target co-occurring symptoms, understanding risk factors of high symptom groups including biosocial mechanisms of co-occurring symptoms, and a longitudinal study examining changes in symptom subgroups over the course of cancer treatments as well as in long-term follow up of cancer survivors.

### Supplementary information

ESM 1 (DOCX 53 kb)

## Data Availability

The data that support the findings are openly available in HealthMeasures Dataverse at https://dataverse.harvard.edu/dataverse/HealthMeasures.
