# Supplementary material for: Comparing symptom clusters in cancer survivors by cancer diagnosis: A latent class profile analysis
Source: Support Care Cancer. 2024 Apr 25;32(5):308. doi: 10.1007/s00520-024-08489-0 (PMC11045444; doi:10.1007/s00520-024-08489-0)
Supplement: Supplementary file 1 — (DOCX 53 kb) [file 520_2024_8489_MOESM1_ESM.docx]

Supplemental Table 1. Characteristics of each of the Latent Classes in Cancer Survivors

1-1. Characteristics of each of the Latent Classes in Prostate Cancer Survivors

| Variables | n (%) | | | | | $\chi^{2}$ |
| --- | --- | --- | --- | --- | --- | --- |
|  | Total  (N =1,060) | Class 1  *WNL*  (n=711, 67%) | Class 2  *Fatigue/SD/Pain*  (n=158, 15%) | Class 3  *Fatigue/SD/Depression*  (n=108, 10%) | Class 4  *All Symptoms*  (n=83, 8%) |  |
| Age at diagnosis (years)  21- 49  50- 64  65 or older | 68 ( 6.4)  485 (45.8)  507 (47.8) | 49 (9.1)  307 (57.2)  181 (33.7) | 8 ( 6.4)  87 (70.2)  29 (23.4) | 6 ( 8.2)  43 (58.9)  24 (32.9) | 5 ( 7.7)  48 (73.8)  12 (18.5) | .047 |
| Sex  Male  Female | 1060 (100.0)  0 ( 0.0) | 711 (100.0)  0 ( 0.0) | 158 (100.0)  0 ( 0.0) | 108 (100.0)  0 ( 0.0) | 83 (100.0)  0 ( 0.0) | N/A |
| Race  White  Black  Asian  Other | 530 (50.1)  301 (28.5)  142 (13.4)  84 ( 7.9) | 378 (53.2)  189 (26.6)  95 (13.4)  48 ( 6.8) | 70 (44.6)  53 (33.8)  22 (14.0)  12 ( 7.6) | 56 (51.9)  21 (19.4)  17 (15.7)  14 (13.0) | 26 (31.7)  38 (46.3)  8 ( 9.8)  10 (12.2) | < .001 |
| Ethnicity  Hispanic  Non-Hispanic | 212 (20.0)  848 (80.0) | 126 (17.7)  585 (82.3) | 42 (26.6)  116 (73.4) | 25 (23.1)  83 (76.9) | 19 (22.9)  64 (77.1) | .053 |
| Marital status  Married/cohabiting  Not married | 761 (72.5)  289 (27.5) | 547 (77.7)  157 (22.3) | 104 (66.7)  52 (33.3) | 71 (66.4)  36 (33.6) | 39 (47.0)  44 (53.0) | < .001 |
| Education  $\leq$ High school  Some college  Undergraduate degree or greater | 376 (35.9)  332 (31.7)  339 (32.4) | 213 (30.4)  224 (32.0)  264 (37.7) | 65 (41.4)  57 (36.3)  35 (22.3) | 50 (46.7)  28 (26.2)  29 (27.1) | 48 (58.5)  23 (28.0)  11 (13.4) | < .001 |
| Employment status  Working  Not Working | 459 (43.4)  598 (56.6) | 347 (48.9)  362 (51.1) | 54 (34.4)  103 (65.6) | 41 (38.0)  67 (62.0) | 17 (20.5)  66 (79.5) | < .001 |
| Stage at diagnosis  I  II  III  IV | 272 (26.5)  591 (57.6)  109 (10.6)  54 ( 5.3) | 194 (28.0)  402 (58.1)  74 (10.7)  22 ( 3.2) | 36 (23.4)  88 (57.1)  19 (12.3)  11 ( 7.1) | 30 (29.1)  51 (49.5)  12 (11.7)  10 ( 9.7) | 12 (15.6)  50 (64.9)  4 ( 5.2)  11 (14.3) | < .001 |
| Cancer treatment history  Surgery  Chemotherapy  Radiation therapy | 483 (46.5)  69 ( 6.8)  408 (39.0) | 330 (47.2)  38 ( 5.6)  253 (36.0) | 64 (41.8)  15 ( 9.9)  74 (46.8) | 50 (47.6)  8 ( 7.8)  48 (45.3) | 39 (48.1)  8 (10.4)  33 (41.8) | .654  .130  .032 |

Note. Abbreviations: N/A = Not applicable; SD = sleep disturbance, WNL = within normal limits.

1-2. Characteristics of each of the Latent Classes in Lung Cancer Survivors

| Variables | n (%) | | | | | $\chi^{2}$ |
| --- | --- | --- | --- | --- | --- | --- |
|  | Total  (N=524) | Class 1  *WNL*  (n=244, 46%) | Class 2  *Fatigue/SD/Pain*  (n=129, 25%) | Class 3  *Fatigue/SD/Depression*  (n=68, 13%) | Class 4  *All Symptoms*  (N=85, 16%) |  |
| Age at diagnosis (years)  21- 49  50- 64  65 or older | 44 ( 8.4)  209 (39.7)  273 (51.9) | 15 ( 6.1)  80 (32.8)  149 (62.1) | 10 ( 7.8)  56 (43.3)  63 (48.8) | 10 (14.7)  22 (32.4)  36 (52.9) | 10 (11.8)  51 (60.0)  24 (28.2) | < .001 |
| Sex  Male  Female | 245 (46.6)  281 (53.4) | 116 (47.5)  128 (52.5) | 53 (41.1)  76 (58.9) | 30 (44.1)  38 (55.9) | 47 (55.3)  38 (44.7) | .221 |
| Race  White  Black  Asian  Other | 365 (69.4)  83 (15.8)  50 ( 9.5)  28 ( 5.3) | 172 (70.5)  34 (13.9)  28 (11.5)  10 ( 4.1) | 90 (69.8)  25 (19.4)  10 ( 7.8)  4 ( 3.1) | 48 (70.6)  10 (14.7)  4 ( 5.9)  6 ( 8.8) | 54 (63.5)  14 (16.5)  9 (10.6)  8 ( 9.4) | .301 |
| Ethnicity  Hispanic  Non-Hispanic | 51 ( 9.7)  475 (90.3) | 16 ( 6.6)  228 (93.4) | 14 (10.9)  115 (89.1) | 8 (11.8)  60 (88.2) | 13 (15.3)  72 (84.7) | .097 |
| Marital status  Married/cohabiting  Not married | 303 (57.8)  221 (42.2) | 159 (65.2)  85 (34.8) | 65 (50.8)  63 (49.2) | 40 (58.8)  28 (41.2) | 38 (45.2)  46 (54.8) | .004 |
| Education  $\leq$ High school  Some college  Undergraduate degree or greater | 228 (43.8)  192 (36.9)  100 (19.2) | 87 (36.0)  86 (35.5)  69 (28.5) | 62 (48.8)  51 (40.2)  14 (11.0) | 32 (47.8)  25 (37.3)  10 (14.9) | 47 (56.0)  29 (34.5)  8 ( 9.5) | < .001 |
| Employment status  Working  Not Working | 118 (22.8)  399 (77.2) | 67 (27.8)  174 (72.2) | 25 (19.5)  103 (80.5) | 19 (28.8)  47 (71.2) | 8 ( 9.8)  74 (90.2) | .004 |
| Stage at diagnosis  I  II  III  IV | 185 (36.3)  73 (14.3)  138 (27.1)  113 (22.2) | 92 (39.0)  32 (13.6)  59 (25.0)  53 (22.5) | 45 (36.0)  19 (15.2)  39 (31.2)  22 (17.6) | 22 (33.8)  9 (13.8)  17 (26.2)  17 (26.2) | 26 (31.0)  13 (15.5)  23 (27.4)  22 (26.2) | .842 |
| Cancer treatment history  Surgery  Chemotherapy  Radiation therapy | 291 (55.7)  307 (59.0)  225 (43.1) | 143 (58.6)  130 (53.3)  99 (40.6) | 76 (59.8)  79 (61.7)  54 (41.9) | 33 (48.5)  46 (68.7)  28 (43.1) | 39 (47.0)  53 (63.9)  43 (52.4) | .128  .069  .304 |

Note. Abbreviations: SD = sleep disturbance, WNL = within normal limits.

1-3. Characteristics of each of the Latent Classes in Non-Hodgkin Lymphoma Survivors

| Variables | n (%) | | | | | $\chi^{2}$ |
| --- | --- | --- | --- | --- | --- | --- |
|  | Total    (N =390) | Class 1  *WNL*  (n=216, 55%) | Class 2  *Fatigue/SD/Pain*  (n=92, 24%) | Class 3  *Fatigue/SD/Depression*  (n=42, 11%) | Class 4  *All Symptoms*  (n=40, 10%) |  |
| Age at diagnosis (years)  21- 49  50- 64  65 or older | 114 (29.2)  129 (33.1)  147 (37.7) | 59 (27.3)  64 (29.6)  93 (43.1) | 26 (28.3)  33 (35.9)  33 (35.9) | 14 (33.3)  15 (35.7)  13 (31.0) | 15 (37.5)  17 (42.5)  8 (20.0) | .163 |
| Sex  Male  Female | 200 (51.3)  190 (48.7) | 111 (51.4)  105 (48.6) | 45 (48.9)  47 (51.1) | 21 (50.0)  21 (50.0) | 23 (57.5)  17 (42.5) | .836 |
| Race  White  Black  Asian  Other | 259 (66.4)  50 (12.8)  50 (12.8)  31 ( 7.9) | 154 (71.3)  23 (10.6)  29 (13.4)  10 ( 4.6) | 51 (55.4)  14 (15.2)  16 (17.4)  11 (12.0) | 30 (71.4)  5 (11.9)  1 ( 2.4)  6 (14.3) | 24 (60.0)  8 (20.0)  4 (10.0)  4 (10.0) | .032 |
| Ethnicity  Hispanic  Non-Hispanic | 83 (21.3)  307 (78.7) | 36 (16.7)  180 (83.3) | 24 (26.1)  68 (73.9) | 10 (23.8)  32 (76.2) | 13 (32.5)  27 (67.5) | .066 |
| Marital status  Married/cohabiting  Not married | 224 (58.0)  162 (42.0) | 135 (62.5)  81 (37.5) | 54 (60.0)  36 (40.0) | 18 (43.9)  23 (56.1) | 17 (43.6)  22 (56.4) | .035 |
| Education  $\leq$ High school  Some college  Undergraduate degree or greater | 137 (35.7)  121 (31.5)  126 (32.8) | 64 (30.2)  72 (34.0)  76 (35.8) | 36 (39.6)  31 (34.1)  24 (26.4) | 13 (31.7)  11 (26.8)  17 (41.5) | 24 (60.0)  7 (17.5)  9 (22.5) | .012 |
| Employment status  Working  Not Working | 184 (48.2)  198 (51.8) | 117 (55.2)  95 (44.8) | 36 (40.4)  53 (59.6) | 20 (47.6)  22 (52.4) | 11 (28.2)  28 (71.8) | .006 |
| Stage at diagnosis  I  II  III  IV | 126 (34.0)  80 (21.6)  52 (14.0)  113 (30.5) | 76 (37.1)  37 (18.0)  27 (13.2)  65 (31.7) | 25 (28.1)  23 (25.8)  12 (13.5)  29 (32.6) | 15 (38.5)  9 (23.1)  6 (15.4)  9 (23.1) | 10 (26.3)  11 (28.9)  7 (28.4)  10 (26.3) | .621 |
| Cancer treatment history  Surgery  Chemotherapy  Radiation therapy | 69 (18.0)  290 (75.3)  101 (26.1) | 40 (19.0)  145 (67.8)  53 (24.8) | 12 (13.0)  79 (87.8)  23 (25.3) | 7 (16.7)  31 (75.6)  16 (38.1) | 10 (25.6)  35 (87.5)  9 (22.5) | .353  < .001  .304 |

Note. Abbreviations: SD = sleep disturbance, WNL = within normal limits.

1-4. Characteristics of each of the Latent Classes in Breast Cancer Survivors

| Variables | n (%) | | | | | $\chi^{2}$ |
| --- | --- | --- | --- | --- | --- | --- |
|  | Total  (N =1,500) | Class 1  *WNL*  (n=864, 57%) | Class 2  *Fatigue/Pain/SD*  (n=282, 19%) | Class 3  *SD/Fatigue/Depression*  (n=161, 11%) | Class 4  *All Symptoms*  (n=193, 13%) |  |
| Age at diagnosis (years)  21- 49  50- 64  65 or older | 611 (40.7)  514 (34.3)  375 (25.0) | 331 (38.3)  290 (33.6)  243 (28.1) | 114 (40.4)  102 (36.2)  66 (23.4) | 76 (47.2)  52 (32.3)  33 (20.5) | 90 (46.6)  70 (36.3)  33 (17.1) | .019 |
| Sex  Male  Female | 0 ( 0.0)  1500 (100.0) | 0 ( 0.0)  864 (100.0) | 0 ( 0.0)  282 (100.0) | 0 ( 0.0)  161 (100.0) | 0 ( 0.0)  193 (100.0) | N/A |
| Race  White  Black  Asian  Other | 715 (47.4)  288 (19.2)  367 (24.5)  128 ( 8.5) | 431 (49.9)  158 (18.3)  216 (25.0)  59 ( 6.8) | 123 (43.9)  57 (20.4)  64 (22.9)  36 (12.9) | 76 (47.2)  27 (16.8)  47 (29.2)  11 ( 6.8) | 85 (44.0)  46 (23.8)  40 (20.7)  22 (11.4) | .019 |
| Ethnicity  Hispanic  Non-Hispanic | 295 (19.7)  1205 (80.3) | 134 (15.5)  730 (84.5) | 29 (18.0)  132 (82.0) | 29 (18.0)  132 (82.0) | 57 (29.5)  136 (70.5) | < .001 |
| Marital status  Married/cohabiting  Not married | 835 (56.2)  650 (43.8) | 537 (62.6)  321 (37.4) | 136 (49.1)  141 (50.9) | 78 (48.4)  83 (51.6) | 84 (44.4)  105 (55.6) | < .001 |
| Education  $\leq$ High school  Some college  Undergraduate degree or greater | 443 (29.9)  493 (33.2)  547 (36.9) | 213 (24.9)  268 (31.3)  376 (43.9) | 101 (36.2)  97 (34.8)  81 (29.0) | 51 (32.3)  54 (34.2)  53 (33.5) | 78 (41.3)  74 (39.2)  37 (19.6) | < .001 |
| Employment status  Working  Not Working | 843 (56.9)  638 (43.1) | 545 (63.7)  311 (36.3) | 138 (49.1)  143 (50.9) | 87 (55.1)  71 (44.9) | 73 (39.2)  113 (60.8) | < .001 |
| Stage at diagnosis  I  II  III  IV | 693 (48.3)  557 (38.8)  154 (10.7)  32 ( 2.2) | 435 (52.0)  302 (36.10  84 (10.0)  15 ( 1.8) | 120 (44.8)  107 (39.9)  30 (11.2)  11 ( 4.1) | 70 (46.4)  63 (41.7)  15 ( 9.9)  3 ( 2.0) | 68 (37.6)  85 (47.0)  25 (13.8)  3 ( 1.7) | .018 |
| Cancer treatment history  Surgery  Chemotherapy  Radiation therapy | 1379 (91.9)  891 (59.4)  875 (58.3) | 809 (94.2)  456 (53.5)  526 (61.4) | 249 (90.2)  195 (69.6)  158 (57.5) | 155 (96.9)  99 (62.7)  92 (57.9) | 166 (86.9)  141 (74.2)  99 (52.9) | < .001  < .001  .152 |

Note. Abbreviations: N/A = Not applicable; SD = sleep disturbance, WNL = within normal limits.

1-5. Characteristics of each of the Latent Classes in Uterine Cancer Survivors

| Variables | n (%) | | | | $\chi^{2}$ |
| --- | --- | --- | --- | --- | --- |
|  | Total  (N =354) | Class 1  *WNL*  (n=178, 50%) | Class 2  *Fatigue/SD/Pain*  (n=119, 34%) | Class 3  *All Symptoms*  (n=57, 16%) |  |
| Age at diagnosis (years)  21- 49  50- 64  65 or older | 83 (23.4)  187 (52.8)  84 (23.7) | 40 (22.5)  96 (53.9)  42 (23.6) | 28 (23.5)  61 (51.3)  30 (25.2) | 15 (26.3)  30 (52.6)  12 (21.1) | .957 |
| Sex  Male  Female | 0 ( 0.0)  354 (100.0) | 0 ( 0.0)  178 (100.0) | 0 ( 0.0)  119 (100.0) | 0 ( 0.0)  161 (100.0) | N/A |
| Race  White  Black  Asian  Other | 178 (50.3)  69 (19.5)  64 (18.1)  43 (12.1) | 100 (56.2)  28 (23.5)  17 (14.3)  30 (16.8) | 54 (45.3)  28 (23.5)  17 (14.3)  20 (16.8) | 24 (42.1)  17 (29.8)  9 (15.8)  7 (12.3) | .015 |
| Ethnicity  Hispanic  Non-Hispanic | 96 (27.1)  258 (72.9) | 35 (19.7)  143 (80.3) | 43 (36.1)  76 (63.9) | 18 (31.6)  39 (68.4) | .005 |
| Marital status  Married/cohabiting  Not married | 166 (47.3)  185 (52.7) | 103 (57.9)  75 (42.1) | 13 (23.6)  42 (76.4) | 13 (23.6)  42 (76.4) | < .001 |
| Education  $\leq$ High school  Some college  Undergraduate degree or greater | 122 (35.1)  116 (33.3)  110 (31.6) | 53 (29.8)  51 (28.7)  74 (41.6) | 39 (34.2)  43 (37.7)  32 (28.1) | 30 (53.6)  22 (39.3)  4 ( 7.1) | < .001 |
| Employment status  Working  Not Working | 170 (48.2)  183 (51.8) | 103 (57.9)  75 (42.1) | 54 (45.8)  64 (54.2) | 13 (22.8)  44 (77.2) | < .001 |
| Stage at diagnosis  I  II  III  IV | 276 (79.3)  14 ( 4.0)  45 (12.9)  13 ( 3.7) | 148 (83.1)  6 ( 3.4)  21 (11.8)  3 ( 1.7) | 88 (77.9)  4 ( 3.5)  16 (14.2)  5 ( 4.4) | 40 (70.2)  4 ( 7.0)  8 (14.0)  5 ( 8.8) | .181 |
| Cancer treatment history  Surgery  Chemotherapy  Radiation therapy | 311 (88.6)  88 (25.3)  115 (33.0) | 158 (89.3)  35 (20.1)  49 (28.2) | 104 (88.1)  34 (28.8)  44 (37.3) | 49 (87.5)  19 (33.9)  22 (39.3) | .919  .065  .148 |

Note. Abbreviations: N/A = Not applicable; SD = sleep disturbance, WNL = within normal limits.

1-6. Characteristics of each of the Latent Classes in Cervical Cancer Survivors

| Variables | n (%) | | | | $\chi^{2}$ |
| --- | --- | --- | --- | --- | --- |
|  | Total  (N = 130) | Class 1  *WNL*  (n=55, 42%) | Class 2  *Fatigue/SD*  (n=30, 23%) | Class 3  *All Symptoms*  (n=45, 35%) |  |
| Age at diagnosis (years)  21- 49  50- 64  65 or older | 93 (71.5)  24 (18.5)  13 (10.0) | 37 (67.3)  12 (21.8)  6 (10.9) | 24 (80.0)  3 (10.0)  3 (10.0) | 32 (71.1)  9 (20.0)  4 ( 8.9) | .718 |
| Sex  Male  Female | 0 ( 0.0)  130 (100.0) | 0 ( 0.0)  55 (100.0) | 0 ( 0.0)  30 (100.0) | 0 ( 0.0)  45 (100.0) | N/A |
| Race  White  Black  Asian  Other | 73 (56.2)  23 (17.7)  17 (13.1)  17 (13.1) | 34 (61.8)  6 (10.9)  7 (12.7)  8 (14.5) | 16 (53.3)  7 (23.3)  5 (16.7)  2 ( 6.7) | 23 (51.1)  10 (22.2)  5 (11.1)  7 (15.6) | .579 |
| Ethnicity  Hispanic  Non-Hispanic | 45 (34.6)  85 (65.4) | 16 (29.1)  39 (70.9) | 6 (20.0)  24 (80.0) | 23 (51.1)  22 (48.9) | .011 |
| Marital status  Married/cohabiting  Not married | 57 (44.5)  71 (55.5) | 31 (57.4)  23 (42.6) | 16 (53.3)  14 (46.7) | 10 (22.7)  34 (77.3) | .001 |
| Education  $\leq$ High school  Some college  Undergraduate degree or greater | 59 (46.5)  43 (33.9)  25 (19.7) | 26 (48.1)  13 (24.1)  15 (27.8) | 10 (33.3)  12 (40.0)  8 (26.7) | 23 (53.5)  18 (41.9)  2 ( 4.7) | .019 |
| Employment status  Working  Not Working | 66 (51.2)  63 (48.8) | 35 (63.6)  20 (36.4) | 18 (60.0)  12 (40.0) | 13 (29.5)  31 (70.5) | .002 |
| Stage at diagnosis  I  II  III  IV | 70 (57.4)  12 ( 9.8)  29 (23.8)  11 ( 9.0) | 34 (65.4)  5 ( 9.6)  7 (13.5)  6 (11.5) | 18 (66.7)  2 ( 7.4)  5 (18.5)  2 ( 7.4) | 17 (41.9)  5 (11.6)  17 (39.5)  3 ( 7.0) | .092 |
| Cancer treatment history  Surgery  Chemotherapy  Radiation therapy | 82 (64.1)  73 (56.6)  78 (60.9) | 36 (65.5)  28 (51.9)  30 (55.6) | 22 (73.3)  15 (50.0)  15 (50.0) | 24 (55.8)  30 (66.7)  33 (75.0) | .296  .236  .054 |

Note. Abbreviations: N/A = Not applicable; SD = sleep disturbance, WNL = within normal limits.

1-7. Characteristics of each of the Latent Classes in Colorectal Cancer Survivors

| Variables | n (%) | | | $\chi^{2}$ |
| --- | --- | --- | --- | --- |
|  | Total  (N =802) | Class 1  *WNL*  (n=534, 67%) | Class 2  *All Symptoms*  (n=268, 33%) |  |
| Age at diagnosis (years)  21- 49  50- 64  65 or old8er | 154 (19.2)  311 (38.8)  337 (42.0) | 84 (15.7)  191 (35.8)  259 (48.5) | 70 (26.1)  120 (44.8)  78 (29.1) | < .001 |
| Sex  Male  Female | 383 (47.8)  419 (52.2) | 268 (50.2)  266 (49.8) | 115 (42.9)  153 (57.1) | .052 |
| Race  White  Black  Asian  Other | 409 (51.0)  184 (22.9)  146 (18.2)  63 (7.9) | 297 (55.6)  110 (20.6)  97 (18.2)  30 ( 5.6) | 112 (41.8)  74 (27.6)  49 (18.3)  33 (12.3) | < .001 |
| Ethnicity  Hispanic  Non-Hispanic | 157 (19.6)  645 (80.4) | 88 (16.5)  446 (83.5) | 69 (25.7)  199 (74.3) | .002 |
| Marital status  Married/cohabiting  Not married | 434 (54.8)  358 (45.2) | 303 (57.4)  225 (42.6) | 131 (49.6)  133 (50.4) | .038 |
| Education  $\leq$ High school  Some college  Undergraduate degree or greater | 343 (43.4)  252 (31.9)  195 (24.7) | 202 (38.3)  177 (33.5)  149 (28.2) | 141 (53.8)  75 (28.6)  46 (17.6) | < .001 |
| Employment status  Working  Not Working | 298 (38.2)  483 (61.8) | 216 (41.1)  309 (58.9) | 82 (32.0)  174 (68.0) | .014 |
| Stage at diagnosis  I  II  III  IV | 188 (24.6)  210 (27.5)  259 (33.9)  106 (13.9) | 131 (25.8)  157 (30.9)  160 (31.5)  60 (11.8) | 57 (22.4)  53 (20.8)  99 (38.8)  46 (18.0) | .002 |
| Cancer treatment history  Surgery  Chemotherapy  Radiation therapy | 687 (87.6)  464 (58.7)  134 (17.1) | 463 (88.2)  284 (53.8)  77 (14.6) | 224 (86.5)  180 (68.7)  57 (22.1) | .496  < .001  .009 |

Note. Abbreviations: WNL = within normal limits.
